# Reduced BRCA1 expression due to promoter hypermethylation in therapy-related acute myeloid leukaemia

**DOI:** 10.1038/sj.bjc.6603392

**Published:** 2006-10-17

**Authors:** A Scardocci, F Guidi, F D'Alo', D Gumiero, E Fabiani, A DiRuscio, M Martini, L M Larocca, M Zollino, S Hohaus, G Leone, M T Voso

**Affiliations:** 1 Istituti di Ematologia, di, Universita' Cattolica Sacro Cuore, L.go A. Gemelli, 1, 00168 Roma, Italy; 2 Anatomia Patologica e di, Universita' Cattolica Sacro Cuore, Roma, Italy; 3 Genetica Umana, Universita' Cattolica Sacro Cuore, Roma, Italy

**Keywords:** BRCA1, hypermethylation, t-AML, therapy-related

## Abstract

BRCA1 plays a pivotal role in the repair of DNA damage, especially following chemotherapy and ionising radiation. We were interested in the regulation of BRCA1 expression in acute myeloid leukaemia (AML), in particular in therapy-related forms (t-AML). Using real-time PCR and Western blot, we found that BRCA1 mRNA was expressed at barely detectable levels by normal peripheral blood granulocytes, monocytes and lymphocytes, whereas control BM-mononuclear cells and selected CD34+ progenitor cells displayed significantly higher BRCA1 expression (*P*=0.0003). Acute myeloid leukaemia samples showed heterogeneous BRCA1 mRNA levels, which were lower than those of normal bone marrows (*P*=0.0001). We found a high frequency of hypermethylation of the BRCA1 promoter region in AML (51/133 samples, 38%), in particular in patients with karyotypic aberrations (*P*=0.026), and in t-AML, as compared to *de novo* AML (76 *vs* 31%, *P*=0.0002). Examining eight primary tumour samples from hypermethylated t-AML patients, BRCA1 was hypermethylated in three of four breast cancer samples, whereas it was unmethylated in the other four tumours. BRCA1 hypermethylation correlated to reduced BRCA1 mRNA (*P*=0.0004), and to increased DNA methyltransferase DNMT3A (*P*=0.003) expression. Our data show that reduced BRCA1 expression owing to promoter hypermethylation is frequent in t-AML and that this could contribute to secondary leukaemogenesis.

BRCA1 is a 1863 amino-acid protein, particularly expressed by actively dividing cells, important for regulation of cell proliferation, DNA repair, the maintenance of genomic integrity and induction of apoptosis in damaged cells (Venkitaraman, 2002). In particular, BRCA1 participates in homologous recombination and nonhomologous end-joining, facilitating repair of double-strand DNA breakages, which frequently occur following antiblastic treatment. BRCA1 is widely expressed by proliferating tissues pointing to a general role in maintaining genomic integrity (Blackshear *et al*, 1998). Mutations of *BRCA1* occur in at least 10% of inherited breast cancers, but are very rare in sporadic breast cancer. BRCA1 expression is repressed through promoter hypermethylation in 11–31% of breast, 5–15% of ovarian cancer and about 60% of pancreatic ductal carcinoma (Catteau and Morris, 2002; Peng *et al*, 2006). The biologic effects of the loss of *BRCA1* gene function caused by promoter hypermethylation and by coding-region mutations in breast and ovarian cancer produce similar microarray patterns of reduced gene expression (Hedenfalk *et al*, 2001).

We were interested in BRCA1 expression in normal bone marrow (BM) and peripheral blood (PB) haematopoietic cells, and in AMLs. In particular, we studied BRCA1 regulation by promoter hypermethylation in *therapy-related* AML (t-AML), as drugs and radiation have been shown to induce DNA hypermethylation, and BRCA1 has an important role in the repair process of DNA damage, induced by these agents (Issa *et al*, 1996; Kennedy *et al*, 2004; Pogribny *et al*, 2004).

## PATIENTS AND METHODS

Our analysis included 133 AML patients (60 females and 73 males, median age 62 years, range 16–85 years), diagnosed between April 1995 and October 2004. The diagnosis of AML was established according to morphology and immunophenotype.

Twenty-one patients had a leukaemia secondary to treatment for a previous malignancy (eight breast cancer, two Hodgkin's lymphoma, three non-Hodgkin's lymphoma, two multiple myeloma, one Waldenstrom's macroglobulinemia, one essential thrombocytemia, one prostate, two thyroid and one lung cancer) and were defined ‘therapy-related AML’ (Table 1). Karyotype was available for 92 patients, 15 of whom had a t-AML. Thirty-eight patients had a normal karyotype, 24 a balanced translocation and 30 other chromosomal aberrations. Controls included normal BM and PB samples. Informed consent was obtained from all patients, according to institutional guidelines.

### RNA and protein expression

We studied BRCA1 mRNA expression by real-time PCR in BM samples of 56 AML patients and in several normal blood cell subsets. Mononuclear progenitor cells were separated from 16 normal BM aspirates using Ficoll density centrifugation and CD34+ cells were isolated using immunomagnetic beads (*n*=8, Miltenyi Biotec GmbH, Bergisch Gladbach, Germany). Mature granulocytes were separated from the PB of four normal donors, following 2-h of incubation with Voluven (Fresenius KABI, Italy), and density-gradient centrifugation on Lympholyte-H (Cederlane, Ontario, Canada). Peripheral blood-monocytes (*n*=7) were separated from lymphocytes (*n*=9) following 1-h adherence in culture dishes.

Mononuclear cells (MNC) were separated from the BM of AML patients at the time of initial diagnosis, using Ficoll and were frozen in RLT Buffer (Qiagen GmbH, Hilden, Germany) for RNA extraction and at −80°C as pellets for DNA and protein extraction. The haematopoietic cell lines HL-60, TF-1, KG1 and the lymphoblastic cell lines Raji and Jurkat (all from LGC-ATCC, Middlesex, UK) were also examined.

RNA was extracted using the RNeasy kit (Qiagen) and was reverse transcribed using random hexamers as reaction primers. Quantitative assessments of cDNA amplification for *BRCA1* (Esteller *et al*, 2000), *DNMT1*, *3A*, *3B* and the internal reference genes *18S* and *cyclin D2* for DNMT were performed by a fluorescence-based real-time detection method (Biorad, Munchen, Germany) and the iQ™ SYBR Green SuperMIX (Biorad). Oligonucleotides used are described in Table 2. Polymerase chain reaction consisted of 3 min at 95°C, followed by 30 cycles at 95°C for 15 s and 60°C for 1 min. To assure the amplicon specificity of each primer set, the PCR products were then subjected to a melting curve analysis. For each PCR, a standard curve was produced, using four consecutive 1:10 dilutions of a positive sample. All samples were run in triplicate. The relative amount of mRNA in the samples was calculated using the following calculation: 
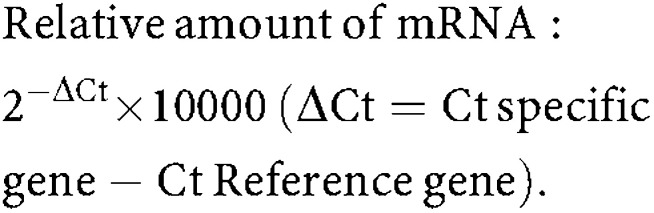


BRCA1 protein expression was analysed by Western blot in 19 AML patients. Proteins were detected by hybridisation with the primary mouse monoclonal anti-BRCA1 (D-9, sc-6954, Santa Cruz Biotechnology, Santa Cruz, CA, USA) and anti-*β*-tubulin antibodies (D-10, sc-5274, Santa Cruz Biotechnology) in 5% bovine serum albumin/1 × TBST/0.1% sodium azide, overnight at 4°C and 1 h at room temperature, respectively.

### Analysis of BRCA1 methylation

We then studied regulation of *BRCA1* promoter hypermethylation in the BM of 133 AML patients, at the time of diagnosis.

The PB of 25 donors (median age 47 years, range 18 –60 years) and 15 normal BMs (median age 39 years, range 23–75 years) were analysed as control. The cell lines HL-60, TF-1, KG1, Raji and Jurkat (LGC-ATCC) were also studied. Patients' and controls' DNA was extracted using DNAzol (Gibco BRL, Eggenstein, Germany), following the manufacturers' instructions. DNA samples extracted from paraffin-embedded tissue from the primary tumour were available for eight patients (four breast cancers, two lymphoproliferative diseases and two lung cancers). Six of these patients had a t-AML, two patients received surgery alone for the primary tumour.

DNA methylation pattern in the CpG island of the *BRCA1* promoter was determined by methylation-specific PCR (MSP), following sodium bisulphite treatment. Methylation-specific PCR, specific for the promoter region of *BRCA1*, was performed with the oligonucleotides in Table 2 (Esteller *et al*, 2000). Polymerase chain reaction was performed using the HotMasterMix (Eppendorf AG, Hamburg, Germany). Ten microliters of each PCR reaction were directly loaded onto a 3% agarose gel, stained with ethidium bromide and visualised under UV illumination. Samples were scored positive only when a distinct band was present on the gel and if *BRCA1* hypermethylation was confirmed in two independent experiments. The sensitivity of this MSP, obtained by diluting a completely methylated sample into a nonmethylated sample, was 10% (data not shown).

### Statistical analysis

Differences in BRCA1 expression levels between two sample groups were analysed using the Mann–Whitney *U* Test. The strength of the association between the expression levels of each DNMT and BRCA1 according to the presence of BRCA1 hypermethylation was calculated by the Spearman rank-correlation coefficient. The Fisher's exact test was used to examine for differences in patients' characteristics according to BRCA1 hypermethylation. A multivariate analysis using the Cox regression model was also performed. Odds ratios (OR) are given within 95% confidence intervals (CI). All computations were performed using the Stata 7.0 software (Stata Corp., College Station, TX, USA).

## RESULTS

### BRCA1 expression

We studied BRCA1 expression in normal haematopoietic cells and AML samples by real-time PCR and by Western blot.

**Figure 1:**
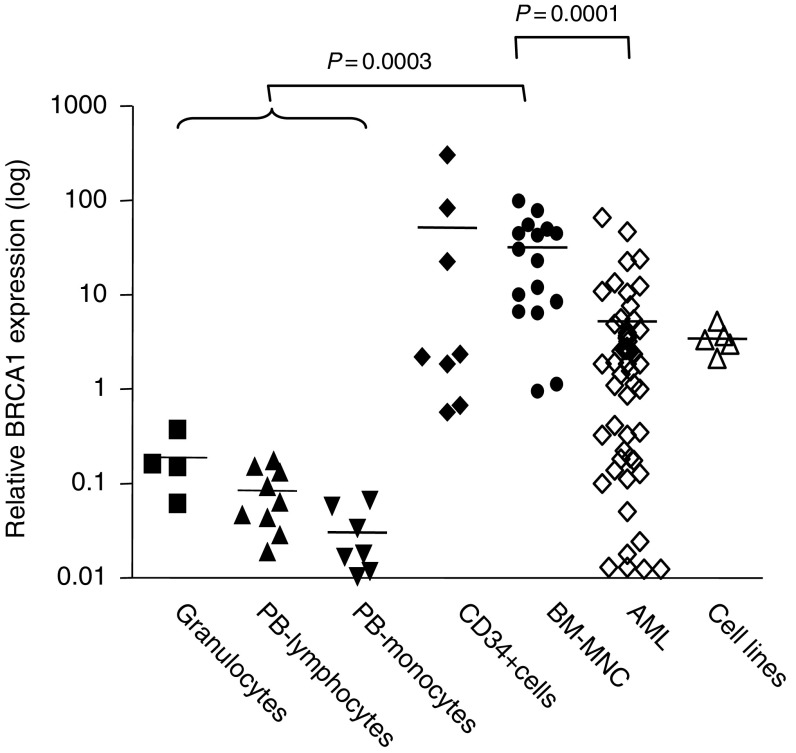
BRCA1 mRNA expression in AML and in normal blood cells. Quantitative assessment of BRCA1 mRNA and the reference gene 18S was performed using a fluorescence-based real-time detection method (RT–PCR) and the dye SYBR Green. Arithmetic means are shown. Normal BM and PB progenitor cells expressed high BRCA1 levels, when compared to mature granulocytes, lymphocytes and monocytes (*P*=0.0001). BRCA1 expression in AML was heterogeneous, similar to the haematopoietic cell lines TF1, HL-60, KG1A, Jurkat and Raji, but lower than that of normal BM (*P*=0.0003).

BRCA1 mRNA was expressed at barely detectable levels in mature granulocytes, monocytes and lymphocytes separated from normal PB (mean relative amount of BRCA1: 0.19±0.07, *vs* 0.03±0.01, *vs* 0.08±0.02, respectively). In contrast, control BM-MNCs and selected CD34+ progenitor cells expressed BRCA1 mRNA at high levels (mean relative amount of BRCA1: 32.3±7 and 52±37, Figure 1).

In comparison to normal BM, BRCA1 expression level was reduced in 56 AML samples (mean BRCA1 relative amount: 5.2±1.5, *P*=0.0001, Figure 1).

**Figure 2:**
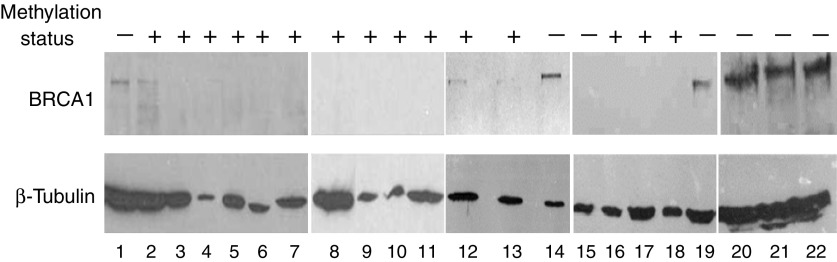
BRCA1 protein expression. BRCA1 protein expression was studied by Western blot in 19 samples of patients with AML (lanes 1–19), and in the TF-1, HL60 and Raji cell lines (lanes 20–22, respectively). Methylation status of the samples is indicated (−: unmethylated, +: methylated for BRCA1).

The BRCA1 mRNA and protein were expressed at high levels by TF1, HL60 and Raji cell lines (Figures 1 and 2, lanes 20–22). BRCA1 protein expression in 19 AML samples was variable, and consistently lower than that of cell lines (Figure 2, lanes 1–19).

### BRCA1 hypermethylation

**Figure 3:**
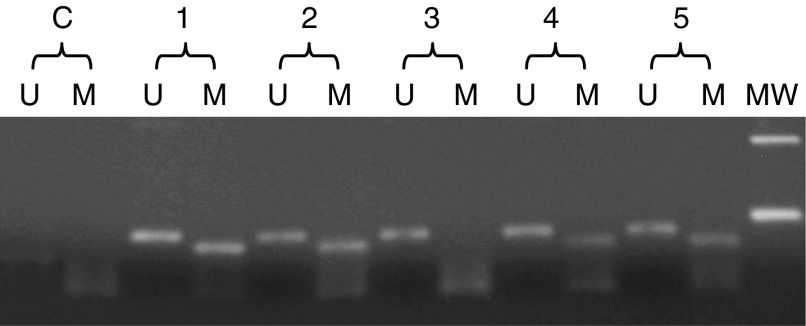
BRCA1 MS-PCR. Samples from patients 1, 2, 4 and 5 present bands for both the unmethylated (U) and methylated (M) PCR-reactions, whereas that from patient 3 is unmethylated. MW is the molecular weight marker (100 bp DNA ladder), whereas C is the negative control, containing water instead of DNA, for ‘unmethylated’ and ‘methylated’ PCR reactions.

As promoter hypermethylation is a frequent event in malignant diseases, leading to reduced tumour suppressor gene expression, we then studied regulation of BRCA1 expression by promoter hypermethylation in AML patients. DNA samples from 133 AML patients were available, and 51 of them (38%) resulted hypermethylated. Fifteen BM and 25 PB samples from healthy individuals resulted unmethylated. Figure 3 shows an example of BRCA1 MSP.

**Figure 4:**
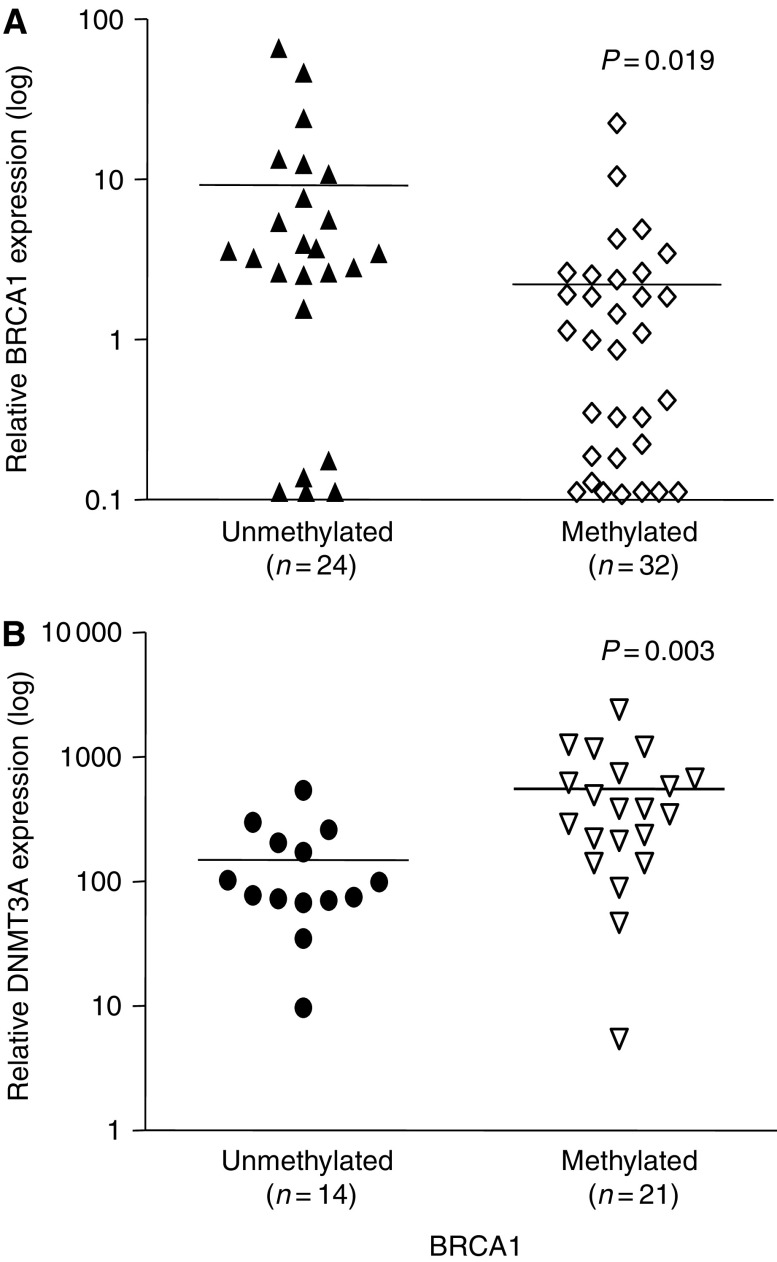
BRCA1 mRNA expression according to methylation status. In patients with AML and BRCA1 hypermethylation, a significant reduction of BRCA1 expression was observed (**A**). On the other hand, hypermethylation was associated to high DNMT3A expression (**B**). The samples analysed for each group are shown. _—_ Indicates mean expression levels.

Looking at the functional significance of *BRCA1* hypermethylation, we found that methylation (*n*=32 AML samples) correlated to reduced BRCA1 expression, when compared to unmethylated samples (*n*=24, mean relative amount of BRCA1: 2.2±0.7 *vs* 9.2±3.4, *P*=0.019, Figure 4A). Similarly, BRCA1 protein expression was reduced in methylated AML samples (Figure 2, lanes 2–13, and 16–18), when compared to unmethylated samples (Figure 2, lanes 1, 14, 15 and 19).

We next analysed whether dysregulation of *BRCA1* methylation in AML could be correlated to DNA methyltransferases expression levels. We studied 35 AML samples, 21 of them were hypermethylated for BRCA1. DNMTs expression in AML samples correlated to each other (DNMT1 and 3A: *r*=0.71, *P*<0.0001; DNMT3A and 3B: *r*=0.52, *P*=0.0013; DNMT1 and 3B: *r*=0.35, *P*=0.04). Median expression of DNMT3A was higher in AML samples with hypermethylated BRCA1 in comparison to unmethylated samples (Figure 4B, *P*=0.003), whereas expression levels of DNMT1 and 3B did not differ according to BRCA1 methylation (*P*=0.22 and 0.07, respectively). There was a moderate negative correlation between DNMT3A and BRCA1 expression (*r*=−0.35, *P*=0.04).

When looking at patient characteristics associated to *BRCA1* hypermethylation, the presence of chromosomal abnormalities was associated to *BRCA1* hypermethylation (*P*=0.026, Table 1).

Furthermore, a significantly higher frequency of *BRCA1* methylation was observed in patients with t-AML (16/21 t-AML, 75%, *vs* 32/112, 32%, *P*=0.0002, OR 7 95% C.I. 2.4–20.7) (Table 1). The Cox regression model showed that t-AML and karyotype were independently associated to *BRCA1* hypermethylation (*P*=0.04 and 0.05, respectively).

DNA from the primary tumour sample was available for eight patients with *BRCA1* hypermethylation and t-AML. Three tumour samples (three of four breast cancers) were hypermethylated for *BRCA-1*, whereas in four patients hypermethylation was present in the t-AML sample only.

## DISCUSSION

We studied expression of BRCA1, which plays a pivotal role in DNA repair, particularly in response to DNA-damaging agents, in AML and in normal haematopoiesis. We found that BRCA1 is expressed at high levels in normal BM progenitor and in CD34+ cells, whereas its expression is reduced in mature PB cells, such as granulocytes, monocytes and lymphocytes. The variable BRCA1 expression may be related to different proliferation rates in the mature compartment when compared to progenitor cells, but also point to a functional role of BRCA1 during the early phases of haematopoiesis, to maintain genomic integrity.

There are only few data on BRCA1 expression in haematologic malignancies. In chronic myeloid leukaemia, it has been shown that expression of the p210 BCR-ABL fusion protein leads to a downregulation of BRCA1 protein expression, owing to post-transcriptional mechanisms (Deutsch *et al*, 2003). In AML samples, we found heterogeneous BRCA1 expression. As leukaemias are among malignancies with the highest methylation density (Melki *et al*, 1999; Costello *et al*, 2000), we studied regulation of BRCA1 expression by promoter hypermethylation. Indeed, we found that 32% of primary AML samples and 75% of t-AML are at least partially hypermethylated at the BRCA1 locus. Hypermethylation of *BRCA1* correlated to reduced mRNA and protein expression, when compared to unmethylated samples, and to high levels of DNA-methyltransferases 3A. High DNMT expression in AML has been reported also by other authors, and is the basis for new therapeutic approaches using demethylating agents (Mizuno *et al*, 2001; Langer *et al*, 2005). In the same line, 60% of pancreatic carcinomas were recently found to be hypermethylated at the BRCA1 gene and the number of methylated genes significantly correlated to DNMT1 protein overexpression (Peng *et al*, 2006).

*BRCA1* hypermethylation has not been previously reported in AML. Using a Southern blot technique, Bianco *et al* (2000) did not find methylation of *BRCA1* in 19 leukaemia samples, including 11 samples from patients with AML. Using MS-PCR, Esteller *et al* (2001) reported no *BRCA1* methylation in 19 leukaemia samples, but did not specify the type of leukaemias studied. The different results of our study could be due to the large number of patients studied, including 21 patients with t-AML, and to the different sensitivity of our MS-PCR. The frequency of *BRCA1* methylation in our AML patients is similar to that of patients with breast, ovarian and pancreatic carcinomas. Other tumour types as colon cancer and hepatocellular carcinomas did not show aberrant *BRCA1* methylation (Bianco *et al*, 2000; Esteller *et al*, 2001). When analysing primary tumour samples of patients with secondary AML, we found *BRCA1* hypermethylation only in breast cancer samples. The number of samples analysed is small, but three of four breast cancers showed *BRCA1* hypermethylation, which might indicate that in breast cancer complicated by a t-AML, the frequency of *BRCA1* hypermethylation is higher than the 11–31% reported in the literature (Catteau and Morris, 2002). As patients treated for breast cancer are at particular risk for t-AML, it will be of interest to study whether *BRCA1* hypermethylation can identify the patients at increased risk for this complication. The loss of BRCA1 function thus appears to be a double-edged sword: it may increase sensitivity to chemotherapy commonly used in breast and ovarian cancer and translate into favourable prognosis, but on the other side, it may trigger secondary leukaemogenesis.

As primary tumours other than breast cancer in our series did not show *BRCA1* hypermethylation, this suggests a role of the cytotoxic treatment in the induction of DNA hypermethylation in t-AML. Accordingly, hypermethylation of several genes has been reported in t-AML (Christiansen *et al*, 2003; Voso *et al*, 2004; Larson and Le Beau, 2005), and ionising radiation has been shown to induce hypermethylation.

None of the five cell lines we analysed were hypermethylated at the *BRCA1* locus and Esteller *et al* (2000) did not find any *BRCA1* hypermethylation in 21 breast cancer cell lines. Reversibility of methylation has been proposed to be an explanation for the rarity of *BRCA1* hypermethylation in cell lines, where restoration of gene expression could confer a selective advantage during establishment of cultures *in vitro* (Catteau and Morris, 2002).

BRCA1 is of particular importance as a regulator of chemo/radiotherapy-induced damage: it accumulates in nuclear foci during S-phase and reassembles into DNA repair-associated foci after DNA damage. The loss of BRCA1 function results in the accumulation of genomic alterations after exposure to DNA-damaging agents. In the same line, we observed a higher frequency of chromosomal translocations and other aberrations in AML patients with *BRCA1* hypermethylation. This association was independent from the type of AML (*de novo vs therapy-related*).

The results of our study lead to the hypothesis that loss of BRCA1 function could be a pathogenetic mechanism in t-AML. Further experimental studies will have to examine the consequences of reduced BRCA1 gene expression in AML on DNA repair and genomic stability.

## Figures and Tables

**Table 1:** BRCA1 methylation status and patients' characteristics

		**Methylated**	**Unmethylated**	
	***n***	***n* (%)**	***n* (%)**	***P***
Age (years)	<60 (60)	20 (33)	40 (67)	0.5
	>61 (73)	31 (42.5)	42 (57.5)	
				
Gender	Females (60)	25 (42)	35 (58)	0.6
	Males (73)	26 (36)	47 (64)	
				
Karyotype (*n*=92)	Normal (38)	8 (21)	30 (79)	**0.026**
	Simple Transl. (24)	9(37.5)	15 (62.5)	
	Complex (30)	15 (50)	15 (50)	
				
Trilineage dysplasia	No (90)	30 (33)	60 (67)	0.1
	Yes (39)	19 (49)	20 (51)	
				
				
Blast cell% (*n*=112)	<50 (39)	17 (43.5)	22 (56.5)	0.3
	>50 (73)	24 (33)	49 (67)	
				
WBC counts (*n*=127)	<30 × 10^9^ l^−1^ (96)	34 (35)	62 (65)	0.4
	>30 × 10^9^ l^−1^ (31)	14 (45)	17 (55)	
				
Type	*De novo* (112)	35 (31)	77 (69)	**0.0002^a^**
	Therapy-related (21)	16 (76)	5 (24)	
				
Primary tumour	Breast (8)	7 (87.5)	1 (12.5)	
	Lymphoproliferative (8)	6 (75)	2 (25)	
	Thyroid (2)	2 (100)	0 (0)	
	Other (3)	1 (33.3)	2 (67)	
				

( ): % of the total number of patients analysed in each group.

a OR: 7; 95% CI: 2.4–20.7.

**Table 2:** Oligonucleotide sequences

**Gene**	**Sequence**	**Fragment length (bp)**	**Reference or gene accession number**
BRCA1-Unmet	5′-TTGGTTTTTGTGGTAATGGAAAAGTGT 5′-CAAAAAATCTCAACAAACTCACACCA	86	Esteller *et al* (2000)
			
BRCA1-Met	5′-TCGTGGTAACGGAAAAGCGC 5′-AAATCTCAACGAACTCACGCCG	76	Esteller *et al* (2000)
			
BRCA1-mRNA	5′-ATGCTGAATGAGCATGATTTT 5′-AGAGTGCTACACTGTCCAAC	352	Esteller *et al* (2000)
			
18S-mRNA	5′-CGTTGATTAAGTCCCTGCCCTT 5′-TCAAGTTCGACCGTCTTCTCAG	136	gi:337376
			
DNMT1-mRNA	5′-GGTTCTTCCTCCTGGAGAATGTC 5′-GGGCCACGCCGTACTG	141	gi:6684524
			
DNMT3A-mRNA	5′-AGGAAGCGCAAGCACCC 5′-ATTGGGTAATAGCTCTGAGGCG	114	gi:18033252
			
DNMT3B-mRNA	5′-CCAGCCCTGCGGCAG 5′-GTTGACGAGGATCGAGTCTTCC	93	gi:18033254
			
CCND2-mRNA	5′-CGCTCACTTGTGATGCCCT 5′-CTGCTCCTGGCAAGCTTTGAG	83	gi:16950656
